# Biotransformation of diclofenac by *Stenotrophomonas humi* strain DIC_5 and toxicological examination of the resulting metabolites

**DOI:** 10.1007/s00253-024-13320-1

**Published:** 2024-10-11

**Authors:** Márton Pápai, Tibor Benedek, Csilla Sörös, Judit Háhn, Zsolt Csenki, Illés Bock, András Táncsics, Balázs Kriszt

**Affiliations:** 1 Department of Molecular Ecology, Hungarian University of Agriculture and Life Sciences, Institute of Aquaculture and Environmental Safety, Páter Károly u. 1, 2100 Gödöllő, Hungary; 2 S.C. Remete Analytica Laboratories S.R.L, Ro-537250 Gyergyóremete, Romania; 3 Department of Food Chemistry and Analytical Chemistry, Hungarian University of Agriculture and Life Sciences, Institute of Food Science and Technology, Villányi út 29-43, 1118 Budapest, Hungary; 4 Department of Environmental Toxicology, Hungarian University of Agriculture and Life Sciences, Institute of Aquaculture and Environmental Safety, Páter Károly u. 1, 2100 Gödöllő, Hungary; 5 Department of Environmental Safety, Hungarian University of Agriculture and Life Sciences, Institute of Aquaculture and Environmental Safety, Páter Károly. u. 1, 2100 Gödöllő, Hungary

**Keywords:** Pharmaceutical, Zebrafish, *Aliivibrio fischeri*, Biotransformation, Nitro-diclofenac, Toxicity

## Abstract

**Abstract:**

The widely used non-steroidal anti-inflammatory drug, diclofenac, detected in increasing concentrations in freshwater ecosystems, is among the most pressing environmental problems today. In this study, the bacterial isolate *Stenotrophomonas humi* strain DIC_5 was capable of degrading diclofenac. It eliminated 75.1% of diclofenac at an initial concentration of 1.5 mg/L after 8 days in the presence of glucose (3.0 g/L). During the process, nitro-diclofenac was identified as a resulting metabolite, whose concentration increased significantly in the bacterial medium from the 7th day of the experiment, while the concentration of diclofenac decreased correspondingly. The ecotoxicological tests on *Aliivibrio fischeri* and zebrafish embryos showed that the bacterial metabolites without diclofenac have a higher toxicity (up to 35.5% bacterial bioluminescence inhibition and 36.7% embryo mortality) than the diclofenac degradation residues (28% and 26.7%, respectively). Based on these results, neither diclofenac nor its degradation products exhibit toxic effects on the test organisms. Conversely, the toxic effect caused by the bacteria was reduced in the presence of diclofenac. Our work highlights the importance of using biotic controls in biotransformation trials, especially when the foreign material is applied in intermediate or environmentally relevant concentration ranges.

**Key points:**

*• Biotransformation of diclofenac by bacteria isolated from a bacterial biofilm.*

*• Biotransformation of diclofenac led to the formation of nitro-diclofenac.*

*• Microorganisms are alternatives for reducing the concentration of diclofenac in water.*

## Introduction

Through human activities, many pollutants are released into the environment, including increasingly prominent drugs and medicinal products. Simultaneously with global drug consumption, the concentration of pharmaceutically active compounds (PhACs) in the environment will continue to increase (Silori et al. 2022). PhACs often have unknown effects on organisms and the environment, as they can be persistent and prone to bioaccumulation, biotransformation, solubility, and even toxicity (Khan et al. 2020, 2021).

Among others, diclofenac (DCF) is an emerging pollutant belonging to the group of non-steroidal anti-inflammatory drugs (Brune & Patrignani 2015). These salts are mainly used in oral pharmaceutical preparations for pain relief, inflammation reduction, and fever relief (Altman et al. 2015). A large amount of diclofenac derives from the pharmaceutical industry, hospitals, and domestic wastewater, and its removal efficiency in sewage treatment plants is low (21–40%); therefore, diclofenac and its metabolites are present in water bodies worldwide with an average concentration between 45 ng/L and 15 µg/L (Beek et al. 2016; Patel et al. 2019; Sathishkumar et al. 2020; Di Lorenzo et al. 2021; Hanif et al. 2020). In the last decade, the behavior, effects, and fate of DCF in the environment have gained scientific attention (Boisseaux et al. 2017; Fu et al. 2020; Parolini 2020; Poddar et al. 2024; Carneiro et al. 2024). The compound’s toxicity has been investigated by several studies on various test organisms, for example, the reduced immune capacity and immune efficiency of the large marsh snail (*Lymnaea stagnalis*) (Boisseaux et al. 2017), and during the examination of water flea (*Daphnia magna*), Japanese medaka (*Oryzias latipes*), and zebrafish (*Danio rerio*), the hatching of embryos was delayed by diclofenac (Oliveira Pereira et al. 2024; Bio and Nunes 2020). Conventional wastewater treatment processes, such as flocculation, sedimentation, precipitation, and activated sludge treatment, have a limited capacity to remove micropollutants in small quantities (Wang et al. 2016). The most sustainable alternatives are methods based on biological and biotechnological solutions using microorganisms to biodegrade drug residues (Moreira et al. 2018; Styloanou et al. 2018). This technique could be used during wastewater treatment, reducing the human and environmental toxicological fate of DCF. Diclofenac shows a high degree of resistance to biological degradation; despite this, several strains of bacteria have been identified that are capable of effectively eliminating the compound using different initial DCF concentrations. *Raoultella* sp. DD4 and KDF8 isolates could efficiently degrade diclofenac at 0.6 and 1000 mg/L concentrations (Domaradzka et al. 2016; Palyzová et al. 2019). In another study, bacterial strains were isolated from wastewater, *Pseudomonas* sp. DCα4 was the most dominant DCF biodegrade, removing 99.8% of DCF (Poddar et al. 2024). In some research, it was observed that bacterial strains could degrade diclofenac as a sole carbon source (Domaradzka et al. 2016; Palyzová et al. 2019). Zur et al. (2021) found that the efficiency of DCF removal is strictly strain-specific and that the primary substrate is not always beneficial. They isolated four new bacteria capable of degradation of DCF in 25 mg/L concentration in the medium. However, extra carbon and energy sources easily cover the energy-demanding steps of enzyme synthesis (Zur et al. 2021). An example of this is *Enterobacter hormaechei* D15 isolate, which has a diclofenac elimination rate of 52% without an external carbon source, and this increased to 82% in the presence of glucose applying 10 mg/L DCF in the medium (Aissaoui et al. 2017); during degradation, the efficiency increased from 35 to 65% in acetate participation (Bessa et al. 2017). The bacterial strain *Labrys portucalensis* F11 was able to degrade 34 µM (6.8 mg/L) diclofenac 100% in 6 days in the presence of acetate (Moreira et al. 2018).

Unfortunately, very little evidence has been shown as regards the metabolic pathways of DCF in microbiological organisms. In other words, there is a gap in our knowledge concerning the identity and the fate of microbial transformation products (TPs) of such successful degradation experiments. It has been shown that there are strong dissimilarities between human and environmental metabolic pathways of DCF. In the human body, 4′-hydroxy metabolite and a glucuronide conjugate are the major TPs of DCF after consumption (Evgenidou et al. 2015; Stierlin and Faigle 1979). Concerning microbial transformation pathways of DCF in the aquatic environment, in addition to hydroxylated metabolites, other dominant forms also occur (Pérez and Barceló 2008; Poirier-Larabie et al. 2016). In the experiment of Poirier-Larabie et al. three transformation products were observed in municipal wastewater under aerobic conditions: 5-hydroxy-DCF, nitroso-DCF (nitroso group on the carboxylic acid moiety), and decarboxylated-DCF (Poirier-Larabie et al. 2016). The identification of TPs after successful degradation is essential, not only to provide risk assessment data on DCF residues in the environment but also for designing effective wastewater treatment technology for DCF removal. In the present work, the kinetics of DCF degradation was studied at 1.5 mg/L initial drug concentration in the media, by the bacterial strain *Stenotrophomonas humi* DIC_5, isolated in the previous research (Pápai et al. 2023). Furthermore, the ecotoxicity of the by-products formed during the degradation of diclofenac was investigated by applying prokaryotic (Tóth et al. 2019) and eukaryotic (Csenki et al. 2019; Garai et al. 2021, 2020) test organisms.

## Materials and methods

### Chemicals and biological reagents

Diclofenac sodium salt was purchased from Sigma-Aldrich (St. Louis, USA) (D6899-10G). Methanol, acetonitrile (UHPLC-MS grade), and formic acid (suprapure, > 98%) were obtained from VWR (Debrecen, Hungary) and Merck KGaA (Darmstadt, Germany), respectively. Water purification was done with a Milli-Q system (Millipore, 18.2 MΩcm at 25 °C). The ingredients for the Bushnell-Haas mineral medium (BHB) (CaCl_2_ 2H_2_O 0.002 g, MgSO_4_ 7H_2_O 0.02 g, NH_4_NO_3_ 1 g, KH_2_PO_4_ 1 g, FeCl_3_ 6H_2_O 0.005 g, H_2_O 1 L) were also obtained from Sigma-Aldrich. Added D ( +)-glucose (≥ 99%, water-free) was obtained from Carl-ROTH (Karlsruhe, Germany).

### Applied bacterial strain and pre-testing of biotransformation

The bacterium selected for the experiment is the *Stenotrophomonas humi* DIC_5 strain, which was isolated from a thoroughly examined bacterial biofilm in the previous work (Pápai et al. 2023). In addition to the test solutions containing diclofenac as the sole source of carbon and energy, metabolic degradation experiments were also set up, which, in addition to diclofenac, contained more easily metabolizable carbon and energy sources, such as glucose. The degradation setting with the highest efficiency was achieved in the presence of glucose (0.3; 3.0 g/L) with an initial diclofenac concentration of 1.5 mg/L in BHB mineral salts solution.

### The experiment

The biotransformation experiment was conducted in four parallels in 100 mL of BHB mineral medium with 1.5 mg/L initial diclofenac concentration. Based on the results of the preliminary biotransformation experiments, glucose at a concentration of 3 g/L was also added to the test solution.

A total of 100 mL aliquots of the test solution were inoculated with 100 µL of bacterial cell suspensions (OD_600_ = 1) obtained in a physiological saline solution. Abiotic controls were also set up, containing all the abovementioned compounds except the bacterial suspension. The tests were incubated for 2 weeks on a rotary shaker at 145 rpm at 28 ± 2 ۜ°C, and two samples were taken per day from each setting (Sutton 2011; Mytilinaios et al. 2012).

### Sampling and preparation for analysis

For each sampling, 2 mL of liquid was taken from the bulk solutions using a medical syringe and filtered using nylon syringe filters (0.22 µm) (ALWSCI Corporation; Zhejiang, China). Every day, two samples were taken 8 h apart for 14 days. The samples were kept at − 20 °C until chemical analyses.

At the end of the biotransformation experiments, the amount of active pharmaceutical ingredients adsorbed to the biomass was determined. Total microcosm bacterial suspensions were centrifuged (2360 g 10 min) and the biomass (pellet) was resuspended in 5 mL of HPLC-grade acetonitrile. In the next step, the resuspended biomass was ultrasonicated three times for 30 s at 20 kHz at 20% amplitude using a Branson Digital Sonifer (Emerson Industrial Automation). The suspensions were vortexed for 30 s in between each sonication stage. Following the ultrasonication process, the samples underwent centrifugation, and the resulting supernatants were filtered using 0.22 µm nylon syringe filters (ALWSCI Corporation; Zhejiang, China) and transferred into 250-mL round-bottom evaporation flasks, using an IKA® RV10 rotary evaporator (Sigma-Aldrich; Budapest, Hungary), the acetonitrile under vacuum at a maximum temperature of 40 °C. The leftovers were then redissolved in 2.5 mL of mineral salt solution. HPLC was used to ascertain the DCF concentration in the solution, as previously mentioned (Benedek et al. 2022).

### Target component analyses by UHPLC-QQ-MS

Target compound analyses were carried out with an ultra-high performance liquid chromatographic system coupled to triple quadrupole tandem mass spectrometry (UHPLC-QQ-MS) using multiple reaction monitoring (MRM) mode in the case of DCF and its TP. Agilent Ultivo UHPLC-QQ-MS operated with electrospray ion source (ESI) in negative mode was used for the quantitative analyses of DCF as well as semiquantitative determination of microbial DCF-metabolite. The ion-source–dependent conditions were the same as those previously published (Gyuris et al. 2024). The system in MRM mode measured the mass transitions of 294.0/250.2 and 294.0/214.0 (DCF) and 339.0/259.0 and 339.0/295.0 (TP). The column was Agilent Eclipse Plus C18 RRHD, 1.8 µm (2.1 × 50 mm), which was maintained at an elevated temperature (40 °C). DCF and its TP were eluted from the column using (A) 0.1% formic acid and (B) 0.1% formic acid in MeOH mobile phases by gradient elution program with 5 min total run time and with the flow rate of 0.4 mL/min. The injection volume was 5 µL.

### Identification of degradation products by (U)HPLC-MS methods

In this study, high performance liquid chromatographic system coupled to quadrupole time-of-flight mass spectrometry (HPLC-Q-TOF–MS) was used for the identification and structure elucidation of DCF metabolites, while further confirmation UHPLC-QQ-MS was applied. By this combination, both selective and sensitive detection, as well as non-target identification, were successfully gained. QTOF is used for the structure elucidation of unknown compounds. Using target analyses with the QQ in MRM mode, the ion ratio of TP can be measured sensitively and selectively, which further confirms the identification. For high-resolution investigations, an Agilent 1100 HPLC system equipped with a binary solvent delivery system and an autosampler was used. Separation was achieved with a Phenomenex Kinetex 2.6 µm, C18, 150 × 4.6 mm chromatographic column. The injection volume was 20 µL. The eluent was 0.1% HCOOH and 5 mM ammonium formate in water (A) and MeOH (B) with a total run time of 18 min and a flow rate of 0.6 mL/min. The LC system was coupled to a quadrupole time-of-flight tandem mass spectrometer (Agilent 6550 iFunnel Q-TOF–MS system) and was used as previously published (Lamfalusy and Soros 2021). A three-step identification protocol was used to discover the possible TPs in the culture media. The “zero” step of the identification was studying the HPLC-QTOF-MS behavior of the parent compound, which can serve as a guideline for the behavior of TPs with similar structures. The isotopic pattern of DCF is typical, showing the presence of two chlorine atoms, which also helped a lot in finding TPs.

(i) For the first step of identification, Mass Profinder (Agilent Technologies, Version 10.0)-assisted data mining helped the fast reveal of possible TPs. The last samples of the 2-week experiment were tested in this way. MS-only chromatograms were prepared from the four parallel abiotic control samples as well as the four parallel treated samples and were imported to the Mass Profinder.
(ii) In the second step of the investigation, the components found by Mass Profinder were examined individually based on the following identification criteria: (1) Be present in all the treated samples, and not in the untreated ones, (2) interpretation of mass spectra should be matched to the structure (accurate mass measurements with an accuracy threshold of 2 mDa), (3) isotope pattern of the parent ion as well as daughter ion (chlorine atom) should be matched with the theoretical calculated, (4) the signal/noise ratio of the extracted ion chromatographic peaks should be bigger than three, and (5) the retention time of the TP should be resulted according to its polarity compared to DCF.
(iii) In the third step of the identification protocol, the retention time and the ion ratio of MRM transitions were investigated by UHPLC-QQ-MS to confirm the identity of TP.

### Ecotoxicological testing

#### *Aliivibrio fischeri* acute bioluminescence assay (Microtox®)

To determine the cytotoxicity of the biotransformation end products of diclofenac, the acute *Aliivibrio fischeri* bioluminescence assay was carried out according to ISO 11348–2 (2007) with a minor modification regarding the dilution solution as described by Tóth et al. (2019). Toxicity was determined based on the inhibition of the luminescence of the cells after 30 min of exposure. A lot of emphasis was put on testing the control conditions, because the culture media, together with the bacteria, which themselves can produce metabolic products, can affect the result of the test. Samples from the biotransformation experiments (abiotic control: BHB solution contains diclofenac and glucose; biotic control: BHB solution contains glucose and bacterial suspension; BHB + glucose: BHB solution contains glucose; DIC_5: BHB solution contains diclofenac, glucose, and bacterial solution) were filtered (0.22 µm cellulose syringe filter), and the NaCl concentrations of the samples were adjusted to 2 (w/w)%. The toxicity of the pure pharmaceutical active compound diclofenac was also determined. Twenty milligrams per milliliter stock solution of diclofenac was prepared in dimethyl sulfoxide (DMSO, CAS 67–68-5, purity ≥ 99.9%, Fisher Scientific) and diluted from 1.00E + 02 to 6.25E + 00 mg/L. Two weight by weight percent NaCl solution was used as solvent control for each test. The dilution solution contained 2 w/w% NaCl for the biotransformation samples and 2 w/w% NaCl and 1 v/v% DMSO (with a final non-toxic concentration in the test at 0.5 v/v%) for diclofenac. The assays were conducted in duplicate, with control and nine concentrations of the test chemicals at a temperature of 15 ± 0.2 °C. Relative bioluminescence was measured using the Microtox® Model 500 Analyzer (SDI) at the beginning of tests and after a 30-min incubation period. The bioluminescence inhibition caused by the biotransformation end-products and diclofenac was expressed as the percentage of the initial concentration that resulted in a 50% reduction in luminescence compared to the control sample. For each sample, the effective concentration (EC50) values were calculated from the concentration–response curves generated using MicrotoxOmni® software (version 1.1, AZUR Environmental Ltd.) after 30 min of contact time.

#### Zebrafish embryo acute toxicity assay

Zebrafish maintenance and egg collection were carried out in accordance with what was previously described by Csenki et al. (2019). The wild-type AB zebrafish used for the experiments were obtained from a recirculation fish culture system (Techniplast S.p.A., Buguggiate, Italy) operated at the Institute of Aquaculture and Environmental Safety of the Hungarian University of Agricultural and Life Sciences. The fish were kept in 14-h light and 10-h dark periods, and the water quality was automatically maintained by the system (25.5 ± 0.5 °C, pH: 7.0 ± 0.2, conductivity 550 ± 50 µS/cm). The fish were fed twice daily with dry pelleted zebra nut feed (ZEBRAFEED, Sparos Lda., Olhão, Portugal) and once daily with live food (*Artemia nauplii*). The day before the tests, the fish were placed in breeding tanks with a partition, five females and three males per tank. On the day of the tests, after the lights were turned on in the laboratory, the partitions were removed according to the microinjection rate to ensure a continuous supply of one-cell stage embryos. Microinjection was conducted as described by Csenki et al. (2019). Injection volumes were 75 µm droplet diameter and corresponded to an injection volume of 0.22 nL, 100 mm to 0.52 nL, 150 mm to 1.77 nL, and 200 mm to 4.17 nL in the case of abiotic control, biotic control, and DIC-5 samples. The toxicity of the BHB + glucose medium was only tested with the largest droplet volume. Each microinjection was performed in three replicates (20 eggs per replicate).

After 120 h, mortality was determined based on the following lethal endpoints: coagulated embryo, absence of somites, and absence of cardiac function. In the tests, surviving embryos were anesthetized in 100 mg/L MS-222 (tricaine-methanesulfonate) solution and then placed in a 4.5% methylcellulose solution containing an anesthetic and oriented on their right side for photography. Brightfield photographs of embryos were taken at × 30 magnification using a Leica M205 FA microscope equipped with a Leica DFC 7000 T camera and Leica Application Suite X software (Leica Microsystems GmbH, Wetzlar, Germany).

## Results

### Biotransformation of DCF by *Stenotrophomonas humi* DIC_5 strain

During the pre-testing experiments, in the test solutions containing diclofenac alone, the degradation was at most 14.3% after 2 months of incubation. The addition of yeast extract, sodium acetate, and sodium citrate to the Bushnell-Haas test solutions did not increase the biological degradation of diclofenac. However, with the addition of glucose (0.5 g/L), a maximum of 25.3% biotransformation was achieved after 2 months of incubation. As a result of increasing the amount of glucose (3.0 g/L), 72.1% diclofenac biotransformation was completed in 4 weeks, which proved to be suitable for further experiments. The biotransformation experiments were performed at an intermediate concentration of DCF (1.5 mg/L).

**Fig. 1:**
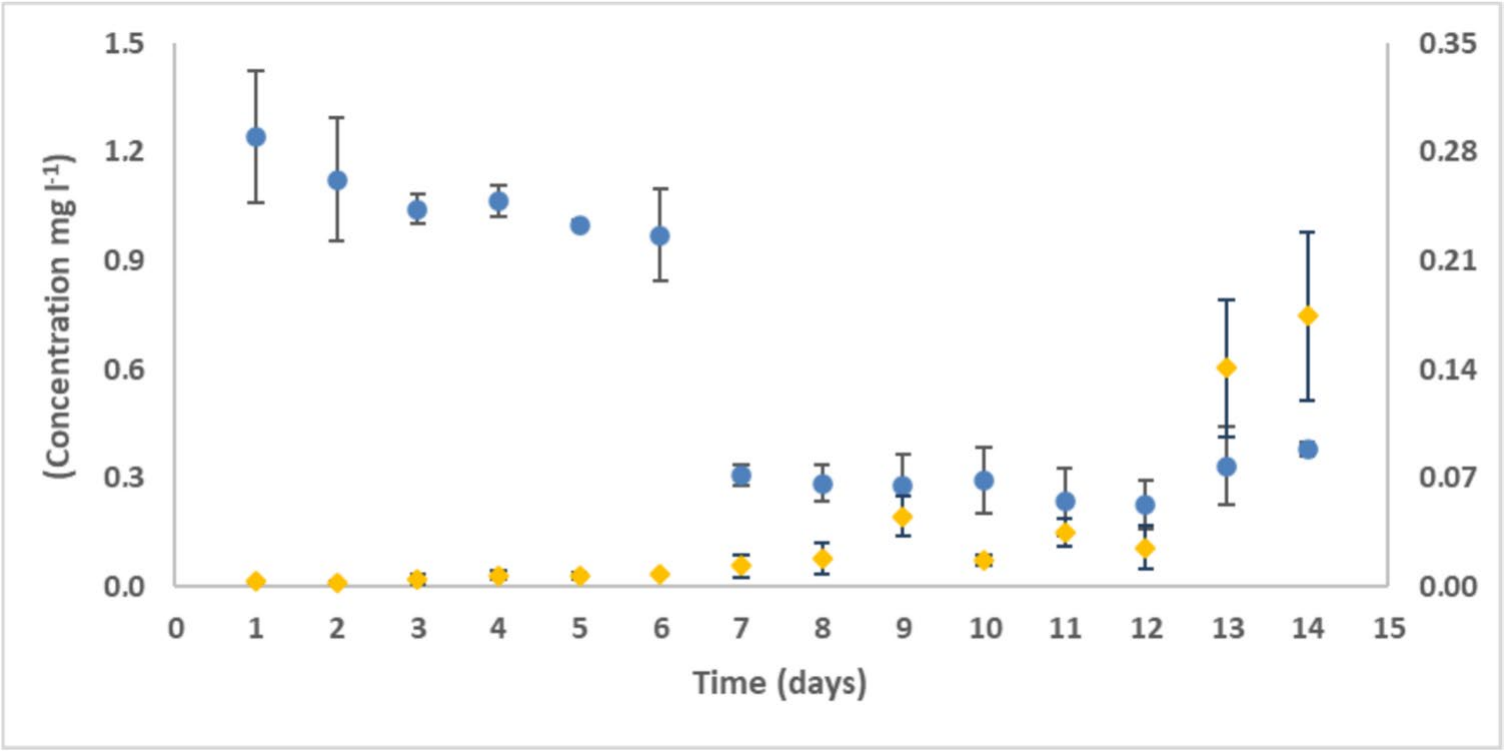
Kinetics of DCF bio by *Stenotrophomonas humi* DIC_5 (blue) (left y-axis) and the change in the concentration of the nitro-DCF metabolite produced during decomposition (orange) (right y-axis)

The main transformation experiment was set up based on the experience attained during the preliminary tests, but the stock solutions were inoculated with a higher bacterial suspension (larger starting quantity). During the first 5 days, no significant changes were observed in the initial concentration of DCF. On the 6th day (Fig. 1, blue dots), a 21.8% reduction of diclofenac was observed. However, on the 8th day, a 75.1% decrease in diclofenac concentration was detected. Meanwhile, the concentration of diclofenac in the abiotic sample did not change compared to the initial value. Based on the biomass test, it can be established that diclofenac was not adsorbed to the cell surface; therefore, the decrease in concentration can be attributed to biological transformation.

### Biotransformation products of diclofenac during *Stenotrophomonas humi* DIC_5 strain biotransformation

**Fig. 2:**
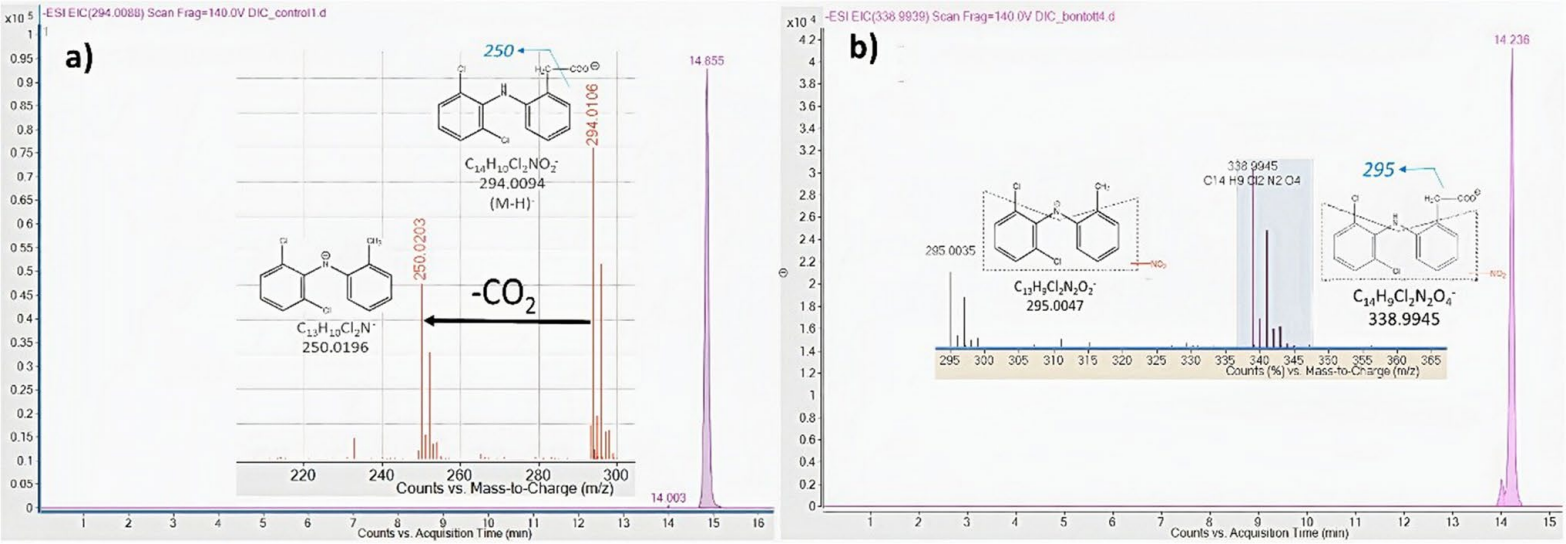
Extracted ion chromatogram (EIC) and mass spectrum of **a** DCF of the abiotic control sample and **b** nitro-DCF of the sample treated by *Stenotrophomonas humi* DIC_5 strain, collected on the 14th day of experiment

The detection and identification of the most relevant TPs are important to predict the environmental impact of DCF. This task requires the application of sophisticated analytical protocol. In this study, HPLC-Q-TOF–MS was used for the identification and structure confirmation of metabolite, while further confirmation and semi-quantitative monitoring of TP during the time of experiment UHPLC-QQ-MS was applied, as a sensitive target component analytical method. The fragmentation of DCF under ( −) ESI condition showed m/z 250.0196 fragment ion, which corresponded to the neutral loss of CO_2_ (44 Da) from the deprotonated molecule ion (M–H)^−^ with 294.0094. Further loss of water (18 Da) was also produced, however with very low intensity (Fig. 2a).

The retention time of DCF in the HPLC-QTOF-MS system was 14.86 min. In the first step of identification of TPs of DCF, 223 of the total findings were recorded by the Agilent Mass Profinder software; however, there was only one chemical entity that fulfilled the identification criteria set by the research team. The molecular ion mass of this TP was measured as 338.9950 which eluted with the retention time of 14.23 from the reverse phase HPLC column. This shows an earlier shift elution from the reverse phase column compared to DCF (14.85 min), which supports the greater polarity of the TP. Generally, the metabolization process usually results in more polar transformation products compared to the mother molecule. The calculated chemical composition of this molecule was C_14_H_10_Cl_2_N_2_O_4,_ which was identified as nitro-DCF. The position of the nitro-group cannot be decided, as the mass resolution of the employed QTOF-MS detector is declared as high as 10,000 FWHM, which is not sufficient enough for discrimination of isobaric interferences. The extracted ion chromatogram (EIC) together with the MS-only spectrum of nitro-DCF is presented in Fig. 2b.

**Fig. 3:**
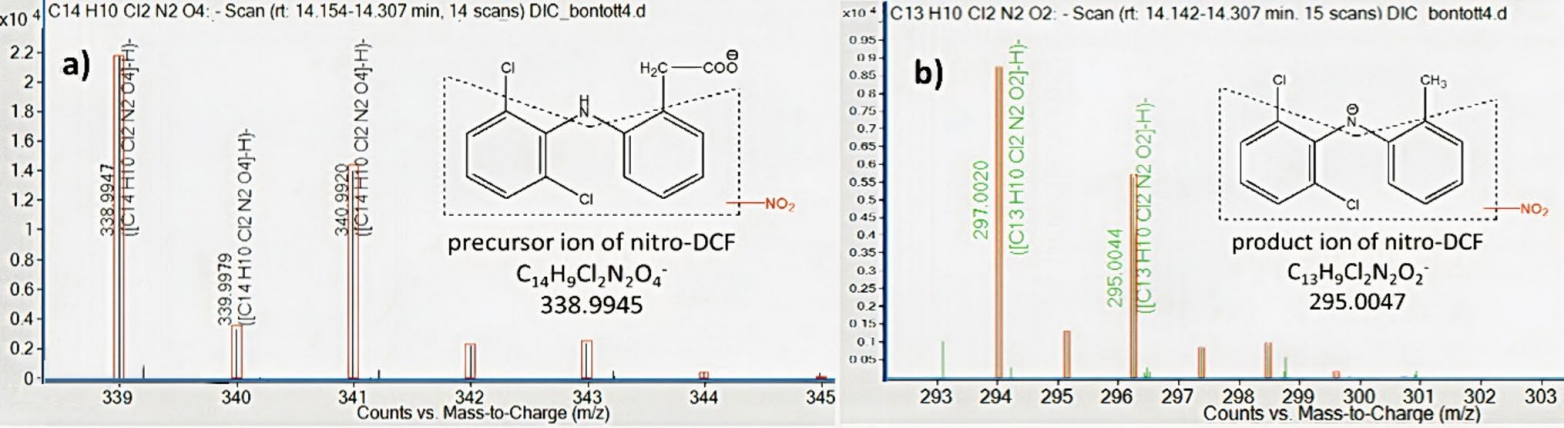
Isotope distribution of **a** precursor and **b** product ion of nitro-DCF in the sample treated by *Stenotrophomonas humi* DIC_5 strain, collected on the 14th day of experiment

The identity of the TP was further confirmed by the interpretation of mass spectra of the chromatographic peak. The MS-only spectrum of nitro-DCF shows a similar fragmentation pathway to DCF. The most intensive fragment ion of 295.0047 was recorded, which is the CO_2_ loss from nitro-DCF (Fig. 2b). The errors of accurate mass, as the difference between calculated mass and measured mass of both molecule ion and fragment were 0.07 mDa and − 0,03 mDa, respectively, which were much below 2 mDa. The isotopic distribution of both the precursor ion as well as of the fragment ion of nitro-DCF matches well with the theoretically calculated one, indicating that the results obtained were reliable (Fig. 3). In addition, double-bond equivalent (DBE_mother_ = 10, DBE_doughter_ = 9) data was also consistent with the proposed structure.

Knowing the fragmentation behavior of nitro-DCF, a sensitive and selective target component analytical method (UHPLC-QQ-MS) was used to examine the change in TP concentration during the experimental period. Both MRM mass transitions of nitro-DCF (339.0/259.0 and 339.0/295.0) could be well detected (S/N > 3) after the 3rd day and show the same ion ratio as DCF (± 30%).

Figure 1 (orange dots) shows the result of semi-quantitative analyses of nitro-DCF in all samples, where the DCF standard was used for calibration. The increase in the concentration of nitro-DCF can be measured from the 7th day, and by the 14th day, it exceeds the concentration of 150 ng/L in the culture media.

The concentration trends of DCF and nitro-DCF show a good correlation. Based on the measured data, cannot assume that nitro-DCF is the only transformation product, however, we did not detect any other TPs in the samples. Nitro-DCF could be easily measured in this amount and can be considered a major metabolite of DCF under such culture conditions.

### Acute toxicity of diclofenac and the biotransformation samples to *Aliivibrio fischeri*

Cytotoxicity of diclofenac as a pure pharmaceutical active compound and the samples from the diclofenac degradation experiment (abiotic control, biotic control, BHB + glucose, DIC_5) was assessed by measuring the reduction in the bioluminescence of *A. fischeri* cells following a 30-min exposure.

**Fig. 4:**
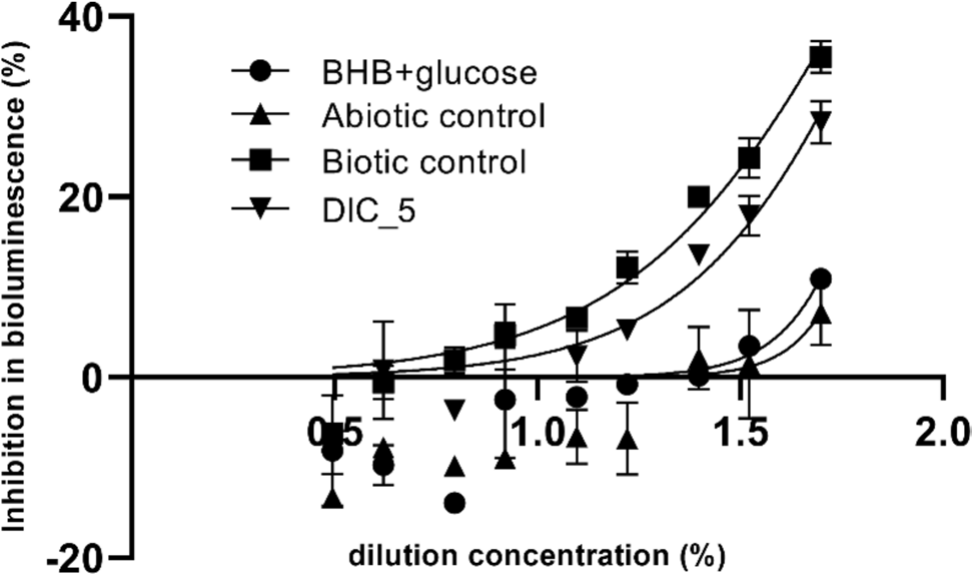
Bioluminescence inhibition (%) in *Aliivibrio fischeri* acute assay after 30 min exposure of samples from diclofenac biotransformation experiments

The effective concentrations of diclofenac as a pure active substance were 7 and 27 mg/L, resulting in a 10 and 50% decrease in bioluminescence, respectively. Correlating to this result, the maximal inhibition of the abiotic control sample containing 1.5 mg/L diclofenac was no more than 7%. The cultivation medium BHB + glucose also resulted in being negligible, with 11% inhibition at the highest applied concentration (Fig. 4). Despite the general sensitivity of this test organism toward diclofenac (Farré et al. 2001; Ra et al. 2008; Ferrari et al. 2003), the results show that neither DCF and its TPs nor the culture media were toxic to the target organism in the applied concentration.

In the second step of the experiment, we examined how the result changes in the presence of bacteria (Fig. 4). An important step is to test the biotic control, which contains the mineral nutrient solution, the additional carbon source (glucose), and the bacterial strain. The latter, only in the presence of nutrients, can also produce metabolites that can be toxic to the test organisms. Based on the results, both the biotic control and DIC_5 samples proved to be slightly toxic to the test organism. The maximal inhibitions of the biotic control and DIC_5 were 35.5 and 28.6%, respectively. The concentrations at which a 50% reduction in luminescence occurred were determined as 73.4% for the biotic control and 89.2% for DIC_5 (Fig. 4).

The results show that *S. humi* strain DIC_5 was able to produce toxic bacterial metabolites. Moreover, the endogenous metabolites of the bacterium seem to be more toxic than the medium which also contains DCF and its TPs (Fig. 4). It can be assumed that the presence of the foreign substance alters the metabolic activity of the bacterium so that it produces less in quantity or different in quality of metabolites. For example, in the work of Palyzová et al. (2019), the proteomic analysis showed that the presence of diclofenac resulted in the upregulation of 24 enzymes associated with the benzoate, catechol, protocatechuate, and ketoadipate pathways (Palyzová et al. 2019). The results draw attention to the necessity of using biotic control in toxicity tests and of carrying out toxicological investigations in the concentration range close to the value found in the environment. Otherwise, the situation may arise that the bacteria used to break down the medicine present in the environment (in micrograms per liter concentration range) turns out to be more cytotoxic than the active substance itself.

### Effects of biotransformation samples on microinjected zebrafish embryos

The biological evaluation of diclofenac degradation by the *Stenotrophomonas humi* DIC_5 bacterium was also performed on zebrafish embryos, using the three-step zebrafish microinjection method previously described by Csenki et al. (2019). Prior to testing the abiotic and biotic controls and the degradation product (DIC_5), the BHB + glucose medium was also tested.

Injection volume is an important factor for post-injection embryo survival, but does not potentially cause egg injury if the injected volume is below 10% of the total yolk volume (Walker et al. 1992), which is 4.2 nL for zebrafish embryos (Schubert et al. 2014). It was expected that the BHB + glucose medium would not cause significant mortality; therefore, it was only evaluated at the largest droplet volume (4.17 nL).

**Fig. 5:**
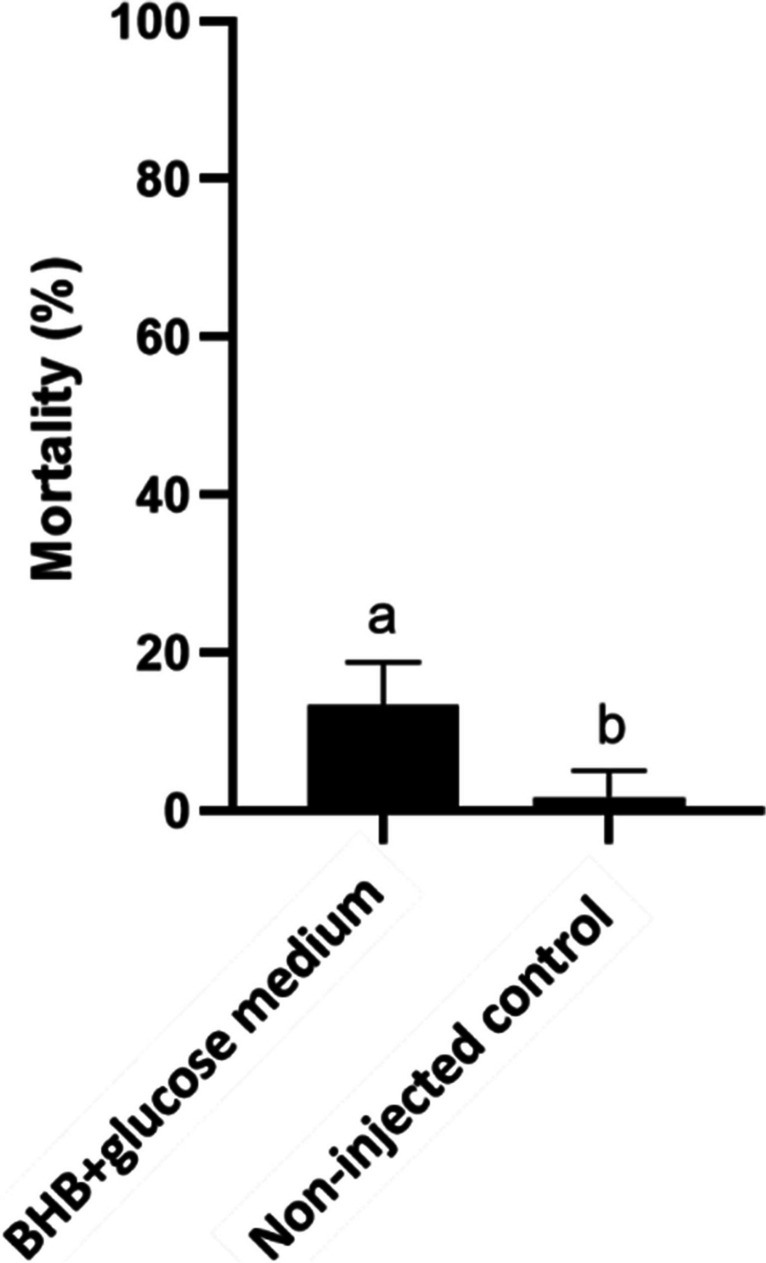
Toxicity of the BHB + glucose culture medium at 4.17 nL injected volume versus non-injected control embryos after 120 h exposure. Different letters indicate significant differences (*p* < 0.05)

The BHB + glucose medium at 4.17 nL injected volume caused significant (13.3 ± 5.4%) mortality versus the non-injected control after 120-h exposure, which means that this culture medium had a relatively low individual toxicity in the applied bioassay (Fig. 5). Since the smaller injection volumes caused proportionally less mortality and malformations for the same substance (Zabel et al. 1995; Csenki et al. 2022) and since the samples from the biotransformation experiment were examined at several concentrations, the mortality results of the other samples were not corrected with the toxicity of the BHB + glucose medium.

**Fig. 6:**
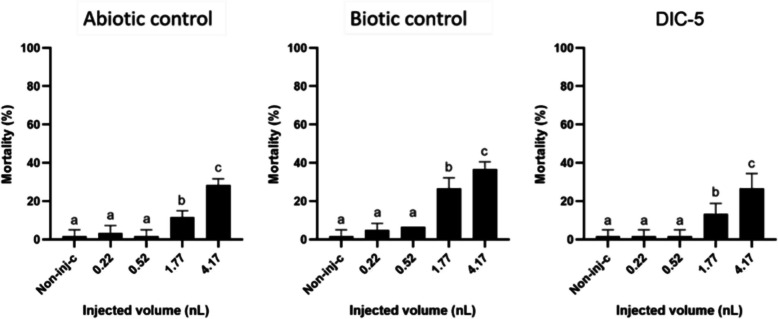
Toxicity of abiotic control (**A**), biotic control (**B**), and DIC-5 (**C**) samples from the biotransformation experiment versus non-injected control embryos after 120-h exposure. Different letters indicate significant differences (*p* < 0.05)

All samples from the biotransformation experiment were tested in four different injected volumes. The results show that the abiotic control, biotic control, and DIC-5 samples caused no significant mortality at the two lowest injected volumes (0.22 nL, 0.52 nL); however, at the two highest injected volumes (1.77 nL, 4.17 nL), all three samples caused a significant dose-dependent increase in mortality (Fig. 6). The mortality at the highest injected volume for the abiotic control was 28.3 ± 3.3%, for the biotic control 36.7 ± 3.9% and for the DIC-5 26.7 ± 7.7%.

**Fig. 7:**
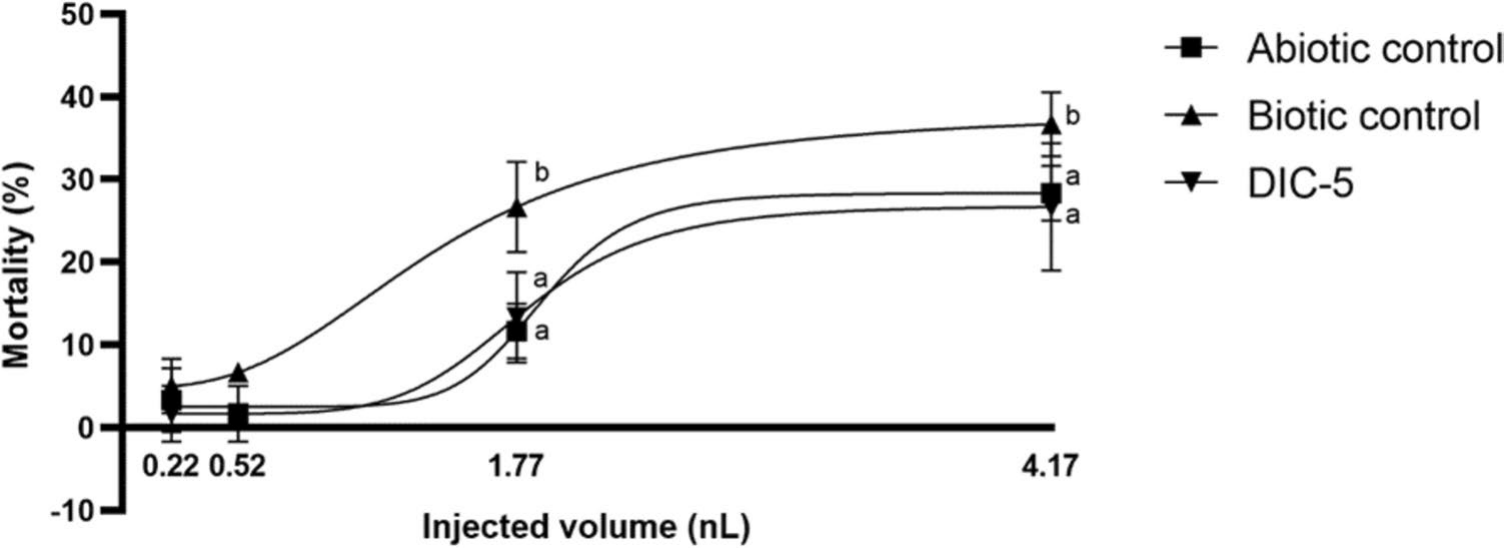
Toxicity of abiotic control, biotic control, and DIC-5 samples from the biotransformation experiment at each injected volume after 120-h exposure. Different letters indicate significant differences (*p* < 0.05)

When comparing the mortality results of the three samples from the biotransformation experiments, the biotic control sample caused significantly higher mortality than the other two samples at 1.77 nL and at 4.17 nL injected volume in the zebrafish embryo tests, which correlates with the results of the *A. fischeri* assay (Fig. 7). The results demonstrate that the *S. humi* DIC_5 bacterium produces toxic endogenous metabolites that—following administration by microinjection—can significantly reduce the survival of zebrafish embryos.

There was no significant difference between the mortality results of the abiotic control and DIC-5 samples at any of the injected volumes, so unlike the *A. fischeri* tests, the zebrafish embryo assay could not distinguish their toxicity from each other nor could the effect of the diclofenac degradation product on mortality be directly determined. However, the results of the zebrafish embryo assays confirm that metabolic activity changes in the bacterium may indeed have occurred in response to diclofenac. The difference in the results of the two models highlights the advantage of using both in vivo and in vitro methods to assess the degradation properties of a toxin-degrading microorganism.

**Fig. 8:**
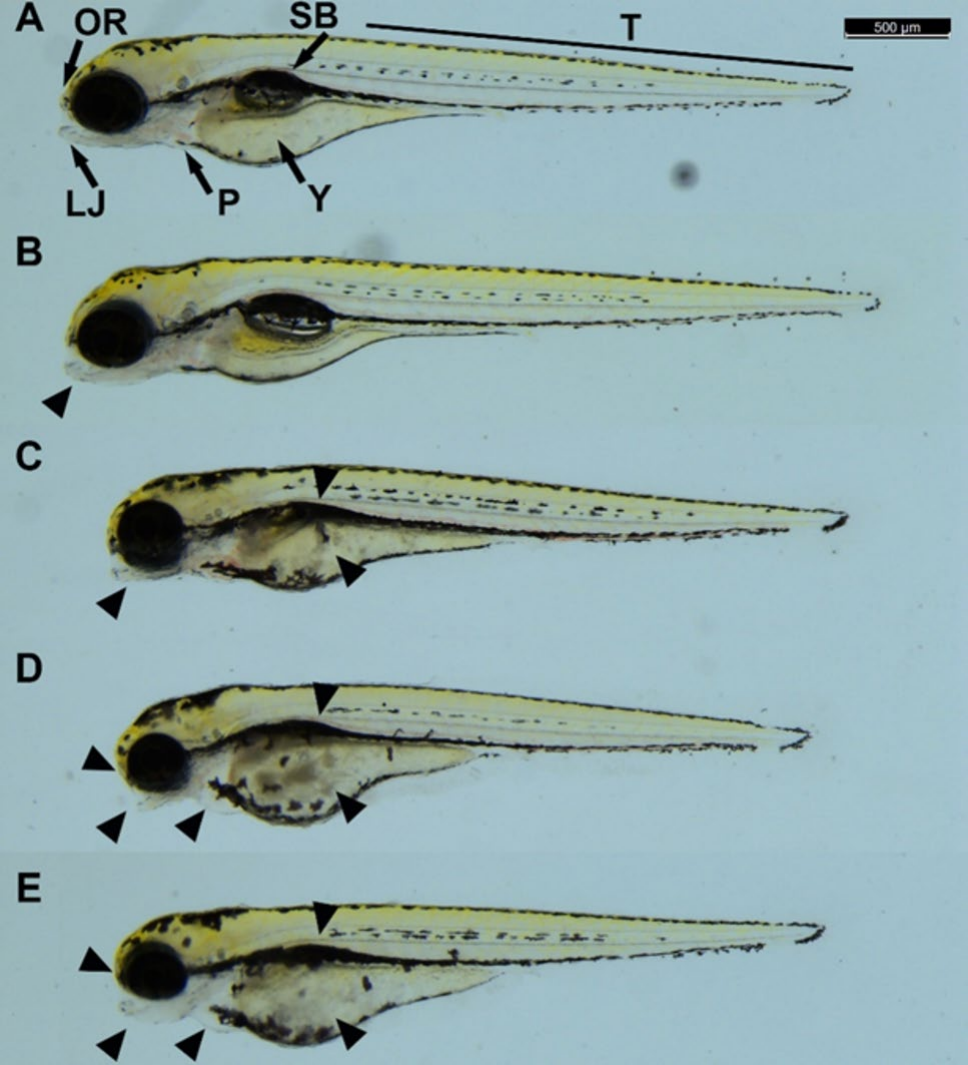
Representative developmental malformations of BHB + glucose medium (**B**), abiotic control (**C**), biotic control (**D**), and DIC_5 (**E**) samples in the zebrafish embryo microinjection assay (control (**A**)). Black arrowheads indicate the location of areas affected by malformations. Abbreviations: OR, olfactory region; LJ, lower jaw; T, tail; SB, swim bladder; P, pericardium; Y, yolk. Scale bar, 500 µm

Beyond mortality, sublethal endpoints were also recorded in embryos at 4.17 nL injected volume after 120-h exposure. In the non-injected control group, no malformations were observed (Fig. 8A). The BHB + glucose medium caused a mild lower jaw malformation of the injected embryos (Fig. 8B).

In fish embryos, nonsteroidal anti-inflammatory drugs (NSAIDs) in general affect heart development, spinal deformity, abnormal yolk absorption, and low hatching rates (Schubert et al. 2014; Chabchoubi et al. 2023). Additionally, the formation of edemas, eye and tail defects, muscle degeneration, shortened body length, trunk curvature, tail malformation, yolk sac abnormalities, and lack of pigmentation were also observed in zebrafish embryos as a result of diclofenac exposure (Hallare et al. 2004; Chen et al. 2014).

From among the symptoms already described for the active ingredient, abnormal yolk absorption, yolk sac abnormalities, and shortened body length were observed in the embryos treated with the abiotic control containing diclofenac. In addition, the lack of swim bladder inflation was observed in all surviving individuals, and a mild deformity of the head region often appeared as well (Fig. 8C).

In the embryos treated with the biotic control (Fig. 8D) and DIC-5 samples (Fig. 8E), similar symptoms were observed, as for the abiotic control, however, the shortened body length was more pronounced. The formation of pericardial edemas, strong underdevelopment of the olfactory region, and characteristic distortion of the lower jaw appeared only in these two samples. These symptoms were most likely caused by the metabolites produced by the studied bacterium *S. humi* strain DIC_5.

## Discussion

Although several studies have successfully performed degradation experiments with different microorganisms at different concentrations of diclofenac (Moreira et al. 2018; Stylianou et al. 2018; Poddar et al. 2024), there is no truly efficient biotransformation protocol for the elimination of DCF, which is evident in the extremely frequent occurrence in wastewater (Wang et al. 2016), drinking water (Sutton 2011), and environmental compartments (Mytilinaios, et al. 2012). Microbial isolate *S. humi* DIC_5 is capable of microbiological transformation of diclofenac at a 1.5 mg/L initial concentration. In general, the members of the genus *Stenotrophomonas* have an important role in the removal of various natural and artificial pollutants, such as polycyclic aromatic hydrocarbons or phenolic compounds (Ryan et al. 2009). Strain LZ-1 can potentially be used for the bioremediation of severe phenol-polluted matrices, for which an indigenous plasmid isolated from the strain is responsible for its degradation (Liu et al. 2007). They play an important ecological role in the nitrogen and sulfur cycle, they are often closely related to plants, helping them grow, and they protect them against some pathogens (Heylen et al. 2007). To this knowledge, this is the first evidence of effective DCF biotransformation by the species *S. humi*. One of the TPs formed during bacterial biotransformation of DCF was identified and confirmed by mass spectrometric methods as nitro-DCF. The test solution contained ammonium nitrate in a nitrogen-rich environment; this type of transformation may be relevant. Nitrogen species (like nitrate, nitrite, and ammonium-ion) are ubiquitous in the natural aquatic environment, and they play an important role in the transformation of organic contaminants (Espinoza et al. 2007). During these processes, nitrifying/denitrifying bacteria are involved which generate reactive nitrogen species, such as NO-radical (Chiron et al. 2010). These species could be responsible for the formation of transformation products, such as nitrosation and nitration derivatives of DCF. The biotic formation of denitrifying derivatives of diclofenac was detected in water/sediment batch experiments under denitrifying conditions by Schubert et al. (2014). Praskova et al. (2011) found that large inputs of ammonium-nitrogen in the wastewater are related to microbial nitrification. The experiment supports the importance of investigating nitro modifications of drug substances as a consequence of biotransformation, which can also occur in wastewater as nitrogen-rich matrices. In the work of Nassef et al. (2010), nitro-DCF was found as the most frequently detected DCF-derivative, being present in 4 out of the 7 wastewater influent samples from Spanish. Ammonia-oxidizing bacteria were tested for removing PP (pharmaceutical pollutants) through the MBR (membrane biochemical reactor). The degradability PPs are NSAIDs, analgesics, antibiotics, and antibacterials. The results showed that ammonia-oxidizing bacteria in the MBR play an important role in the removal of PPs (Park et al. 2017).

In this study, the increase in the concentration of nitro-diclofenac was followed by a decrease in the concentration of diclofenac, with a significant change in concentration from the 7th day.

Based on the results of ecotoxicological studies using *A. fischeri* and zebrafish embryos, it can be concluded that DCF and the secondary products from the biotransformation of diclofenac are practically nontoxic to the test organisms in the concentration range. Surprisingly, the bacterial metabolite products, without the presence of foreign material (DCF) were more toxic to the test organism than the DCF alone or the biotransformated samples. This means that the samples from before biotransformation caused significantly higher mortality than the samples from after the biotransformation process. Alternatively, the toxic effect caused by the bacteria was reduced by the presence of the diclofenac signal. Sublethal endpoints typically accompanying DCF exposure of zebrafish embryos were observed in this study as well, e.g., abnormal yolk absorption, yolk sac abnormalities, shortened body length, uninflated swim bladder, head deformation, shortened body length, edemas, and mandibular deformations. All of these symptoms have been previously described following DCF treatment; no specific sublethal responses were observed in this study (Praskova, et al. 2014). In these studies, using *E. coli* and *Chironomus aprilinus*, the toxicity of AOP by-products was compared with selected biodegradation metabolites of DCF. The results obtained indicate higher toxicity, especially of the by-products formed during UV/H2O2 treatment and ozonation (2,6-dichloroaniline, 2,5-dihydroxyphenylacetic acid) than 4′-OHDCF, 5-OHDCF, and DCF. In particular, evidence was found for higher toxicity of a mixture of diclofenac and chlorogenic acid in *E. coli* (Matejczyk et al. 2020).

Several authors have extensively studied the use of bacteria in wastewater treatment (Shah and Shah 2020). Wider measures should be taken to increase public awareness of the proper management of pharmaceutical residues in wastewater (Matejczyk et al. 2020). Several methods can be combined for the treatment of complex effluents. This could lead to a breakthrough in pharmaceutical wastewater treatment, thus contributing to water sustainability. As regards technical solutions, bio-processes need to be optimized (Eniola et al. 2022). For wastewater containing pharmaceuticals and their metabolites (hospital and pharmaceutical wastewater), additional treatment methods should be implemented. Consideration should be given to the formation of toxic metabolites during the degradation of the drug. This raises the need for additional systems designed for the subsequent removal of toxic intermediates (Matejczyk et al. 2020). Further studies focusing on energy requirements, including operating costs, should be carried out to compare more thoroughly the advanced treatment technologies (Eniola et al. 2022).

## Data Availability

Data will be made available on request. The next bacterium strain, received from Márton Pápai (on behalf of the Hungarian University of Agriculture and Life Sciences, Institute of Aquaculture and Environmental Safety, 2100 Gödöllő, Páter K. u. 1., Hungary) has been accessioned into the National Collection of Agricultural and Industrial microorganisms under the following accession number: Strain designation: *Stenotrophomonas humi* DIC_5Accession number: NCAIM B.02690. The abovementioned strain will be available without restrictions according to the MTA of the National Collection of Agricultural and Industrial Microorganisms.
